# How to Keep Well

**Published:** 1903-08

**Authors:** 


How to Keep Well. An Explanation of Modern Methods of Preventing Disease. By Floyd M. Crandall, M. D., Twelve mo, pages 528. New York: Doubleday, Page & Company. 1903.

This author has written a book with a purpose, and that not solely or chiefly the one of giving instruction in hygiene; indeed, his book might be called a brief of modern medicine itself. To quote from the preface, he has endeavored to tell: "How medicine has risen from mysticism, supernaturalism, and superstition, and, having gradually emerged from the thralldom of astrology upon the one hand and theology upon the other, now stands forth in the foremost rank of the sciences: why it is worthy of acceptance rather than the crude systems of quackery and pseudo-science which one after another arise only to sink into obscurity; to set forth our knowledge regarding the prevention of disease and to show what advances have been made in recent years in our ability to prolong life and to prevent suffering, disease and death."

Dr. Crandall has carried out his endeavor in a commendable manner. The style is neither so simple as to be devoid of interest, nor so technical as to discourage the lay reader. It is written for the intelligent. All physicians deplore, not alone from selfish motives, the spread of pseudo-science and of quackery. The reasonable way to combat this is by the diffusion of knowledge, and to the mind of the reviewer there is no book which is so well adapted to this end as the one under discussion. It is worthy of a wide perusal and might well be introduced into secondary schools to supplement the course in physiology. M. J. F.

